# Individual-, Family-, and School-Level Ecological Correlates With Physical Fitness Among Chinese School-Aged Children and Adolescents: A National Cross-Sectional Survey in 2014

**DOI:** 10.3389/fnut.2021.684286

**Published:** 2021-08-25

**Authors:** Yanhui Dong, Manman Chen, Li Chen, Bo Wen, Yide Yang, Zhenghe Wang, Yinghua Ma, Yi Song, Jun Ma, Patrick W. C. Lau

**Affiliations:** ^1^School of Public Health, Institute of Child and Adolescent Health, Peking University, Beijing, China; ^2^National Health Commission Key Laboratory of Reproductive Health, Peking University, Beijing, China; ^3^School of Medicine, Hunan Normal University, Changsha, China; ^4^Department of Epidemiology, School of Public Health, Southern Medical University, Guangzhou, China; ^5^Department of Sport, Physical Education and Health, Hong Kong Baptist University, Hong Kong, China; ^6^Laboratory of Exercise Science and Health, BNU-HKBU United International College, Zhuhai, China

**Keywords:** physical fitness, ecological correlates, children and adolescents, cross-sectional survey, China

## Abstract

**Introduction:** Few studies have examined the association between the individual-, family-, and school-level ecological correlates and physical fitness among Chinese children and adolescents, which is the purpose of the present study.

**Methods:** A total of 157,168 children and adolescents, 10-18 years of age, with physical fitness data in 2014, participated in the study. Physical fitness was assessed, using six tests: forced vital capacity, standing long jump, sit and reach, body muscle strength, 50-m dash, and endurance running. Scores were aggregated to form a summary physical fitness indicator (PFI), which was then classified into five levels: low, low-middle, middle, middle-high, and high. Each option of individual-, family-, and school-level measures were constructed into a positive or negative correlate of physical fitness and then accumulated as a composite ecological score.

**Results:** Among the 20 individual-, family-, and school-level correlates, 18 were found to be significantly associated with PFI, with high PFI levels being correlated with the positive group of correlates and low PFI levels correlated with the negative group of correlates (*p* < 0.05). High scores of ecological correlates were associated with a high level of PFI [adjusted odds ratio (OR) = 1.06, 95% CI: 1.06, 1.07)] whereas low scores were associated with a low level of PFI (OR = 0.95, 95% CI:0.95, 0.95). The ecological correlates at the individual, school- and family-levels were shown to be significantly related to high PFI among Chinese children and adolescents aged 10-18 years with different ORs of 5.97 (95% CI: 5.51, 6.47), 3.94 (3.66, 4.24), and 1.25 (1.19, 1.31). The PAR% of 20 cumulative factors effects due to the negative and positive responses for low PFI levels were 35.9 and 16.1%, and, for high PFI levels, were 15.3 and 24.1%, among Chinese children and adolescents, respectively. Compared with the correlates at family and school levels, the correlates at individual levels had the largest PAR%.

**Conclusion:** Individual-, family-, and school-multilevel factors had a significant cumulative association with either improving or worsening aspects of physical fitness. Individual level factors remain at the core of physical fitness improvement. Comprehensive policies and measures are urgently needed to enhance the physical fitness of Chinese children and adolescents through involving further individual and environmental factors.

## Introduction

Strengthening the physical fitness of children and adolescents is widely recognized as an important means to improve their health and help them stay physically active and healthy during adulthood (1, 2). The concept of physical fitness involves several core components, including muscular strength, power, endurance, cardiorespiratory fitness, flexibility, and motor ability (3), and previous studies have combined these components into an overall physical fitness indicator (PFI) (4, 5). Evidence to date suggests that low levels of physical fitness, such as reduced aerobic capacity, poor respiratory, or pulmonary function, contribute to the development of cardiovascular diseases in both children and adults (6–8). On the other hand, high levels of physical fitness have been shown to be related to cardiovascular health status, including the low risks of hypertension, diabetes, and other clustered cardiovascular metabolic disorders in child and adolescent populations (9, 10).

Given decades of rapid economic developments, which have resulted in major demographic shifts, lifestyle, and nutritional changes, China has followed global trends in exhibiting steady deteriorating levels of physical fitness among school-aged children and adolescents, particularly in the domain of cardiorespiratory fitness (11–13). Consistent with the evidence shown in other countries, there are many salient factors across individual, family, and school levels that may have contributed to the deterioration of physical fitness in Chinese children and adolescents. Among multilevel ecological factors, individual behaviors play an important role in shaping the physical fitness of a person. For example, sedentary behaviors, smoking, unhealthy dietary patterns, increased body weight, and lack of sufficient physical activity (PA) lead to low levels of physical fitness in children and adolescents. Conversely, high levels of PA and low levels of sedentary behaviors, such as screen time for leisure, contribute to a healthy profile (12, 14). Studies of correlates of physical fitness have also shown a positive association between family support factors (e.g., parental support, encouragement, accompaniment, financial support of PA of children) and increased levels of participation in PA among children and youth (15–17). In addition, environmental features in school settings, such as availability and accessibility of sports facilities, the intensity of the physical education (PE) curriculum, or the academic burden, have been shown to either facilitate or impede PA or physical fitness in children and adolescents (18, 19).

Although prior studies have examined various correlates of PA at individual, family, school, even community levels (20–23), few of them have taken an integrated, ecological approach to understand the cumulative associations and separate relationship between multilevel correlates and physical fitness among children and adolescents (24). Therefore, the objective of this study was to examine the association between physical fitness and ecological correlates (individual-, family-, and school-level factors) of physical fitness, using the 2014 survey data.

## Method

### Study Design and Participants

Data from this study were derived from the 2014 Chinese National Survey on Students' Constitution and Health (CNSSCH), which was first conducted in 1985 and has been investigated every 5 years since then. The 2014 CNSSCH was jointly launched by the Ministry of Education, the Ministry of Health, the Ministry of Science and Technology, the State of Nation Affairs, and the State Sports General Administration of the People's Republic of China in order to investigate the constitution and health of children and adolescents. It has been the largest nationally representative survey so far and comprised the content of physical fitness and social ecological correlates of Chinese children and adolescents 7-18 years of age. The sampling framework and methods of CNSSCH in 2014 were the same as they were in 1985 when they were first conducted. Details of the CNSSCH design have been described in the previous studies (12, 13). In brief, the CNSSCH used a multistage stratified cluster sampling design that included 31 Chinese provinces, excluding Hong Kong, Macau, and Taiwan. In stage 1, Han children and adolescents from the other 30 provinces were included in the study because the survey in Tibet only included ethnic Tibetan children and adolescents. The Han children and adolescents in each province, except Tibet, were stratified by three levels of prefecture cities (i.e., upper, moderate, and low) based on their socioeconomic status (SES). In Stage 2, in each selected city of the three levels of prefecture cities, children and adolescents were stratified by urban and rural areas on the basis of their residence location. In Stage 3, within each of these stratified areas, schools with students aged 7-18 years attending primary, middle, and high schools were randomly selected in 1985 and kept uniform subsequently. In Stage 4, from these schools, all the students from the randomly selected classes by grade were included in the survey. Following the administration of the study inclusion and exclusion criteria and informed consent (25, 26), at least 50 Han students of 7-18 years of age were included in each selected city of the three levels of prefecture cities. In our study, a total of 157,168 children and adolescents 10-18 years of age, who completed the 2014 questionnaire investigation of the CNSSCH, were included.

### Measures of Physical Fitness

#### Quality Control of Measurement

In 2014, the participants at each survey site underwent a physical fitness test battery, (described below) performed in accordance with a standardized protocol. All physical fitness tests were implemented by trained PE teachers who passed their required measurement training examination. As a safety precaution, a school medical doctor was present on the day of testing. A project supervisor monitored the physical fitness testing and provided the necessary guidance to ensure assessment fidelity at each survey site.

#### Items of Measurement

The following six physical fitness measures were (a) forced vital capacity (FVC), (b) standing long jump (SLJ), (c) sit and reach (SR), (d) body muscle strength (BMS), (e) 50-m dash, and (f) endurance running. FVC, SLJ, SR, and 50-m dash were measured among children and adolescents aged 10-18 years. In accordance with the age and sex of children, BMS was evaluated by oblique body pull-ups (among boys aged 10-12 years) and pull-ups (among boys aged 13-18 years) and 1-min sit-ups (among girls aged 10-18 years). Endurance was evaluated by a distance run (a 50 m × 8 shuttle run for both boys and girls aged 10-12 years; a 1,000-m endurance run for boys aged 13-18 years; and an 800-m endurance run for girls aged 13-18 years). Instruments used in the fitness tests included electronic spirometer for FVC and electronic SRfleximeter, and they were calibrated before use and administered consistently at each survey site. Almost all students in one selected school completed their fitness tests on the same day. The sample size and the proportion of the students in each test item are shown in Supplementary Table 1.

#### Calculation of PFI

Taking differences in age and sex into account, we calculated sex- and age-specific standardized values for each of the physical fitness components. Based on the reference population with median value and standard deviation (SD) (4, 12), *Z* scores for each component were calculated as an individual item value minus the median, divided by the SD for the age and sex of a child in the reference population. PFI was calculated by summing the standardized values for each of the six items expressed as: *PFI* = *standardized values of FVC* + *SLJ* + *SR* + *BMS* + *(a*−*50-m dash)* + *(-endurance run)*, with high values indicating good physical fitness. On the basis of PFI percentile values established in the 1985 reference population (12), we classified the PFI values into five categories: low level (<20th), low-middle level (≥20th and <40th), middle level (≥40th and <60th), middle-high level (≥60th and <80th), and high level (≥80th).

### Questionnaire Survey

#### Questionnaire Content and Categories

The project team in the 2014 survey consisted of researchers, personnel from education departments, and principals of the survey schools. The researchers developed the study questionnaire, formed the survey guidelines, and conducted data collection training of PE teachers. Guided by the ecological framework and prior research (27, 28), the team drafted an initial version of the questionnaire through a thorough review of the literature on PA and physical fitness and multiple iterations of item refinement and revisions. This process was repeated until consensus was reached among the members of the project team. The final version of the questionnaire consisted of 20 items that involved individual-, family-, and school-level factors that are relevant to the understanding of PA and fitness of children and adolescents. At the individual level, 10 items were constructed, which included daily sleep duration, breakfast, milk drinking, egg eating, TV screen time, total screen time, homework time, running exercise, PE preference, and PA time. At the family level, two items were developed that included parental PA support to their children/adolescents and the PA preference of parents. At the school level, there were eight items that involved the questions about the frequency of school PE, PE environment, PE effect on students, school recess exercise, attitudes toward school recess exercise, school PA preference of students, school sports competition, and the academic workload. The description of variables regarding this study is shown in Supplementary Table 2.

#### Collection Procedure of Questionnaire

The survey measures and methods have been described in the previous study (29). The field investigators were trained to administer the questionnaire, which took place during school hours. Before the administration, the investigators had described the content of the survey and allowed sufficient time for questions to ensure that the children and adolescents had a good understanding of the survey. Appropriate assistance and guidance were provided if needed. The questionnaire for each student was collected with paper and pencil. The project team members organized personnel to double input all the questionnaires, and combined data information of each province. The survey was completed in ~30 min in each school. All the survey items were checked for completeness and accuracy by the field investigators and project supervisors. The overall survey response rate was 99%. The internal consistency of the survey was modest, as indexed by the Cronbach's α reliability coefficient of 0.68, 0.71, and 0.76 at individual-, family-, and school-level factors. For analysis purposes, the research team classified the survey responses into two categories: positive responses (in support of physical fitness) and negative responses (not in favor of physical fitness). Conceptually, the positive response category represents responses that facilitate the development or improvement of physical fitness, whereas the negative response category represents responses that impede the promotion of physical fitness.

In addition, demographic information, such as age, sex, urban and rural areas, and family prefecture cities, was collected in the survey. The classification of urban and rural areas was defined by the household registration system within the geographical location of the participants. This method was established in 1985 when the first survey was conducted (12). The three levels of prefecture cities (i.e., upper, moderate, and low) in each province were defined by SES, which included regional gross domestic product, total yearly income *per capita*, average food consumption *per capita*, the natural growth rate of population, and the regional social welfare index (13).

### Statistical Analysis

In terms of general statistical analyses, descriptive statistics was used to describe the demographic characteristics of the study population in CNSSCH. Chi-square tests were used to examine between-group differences in categorical variables. To examine the correlates of physical fitness, we analyzed the associations between physical fitness (PFI) and single correlate (each of the survey items assessed in Supplementary Table 2), and then with multiple correlates. In the single correlate analysis, radar maps were conducted to compare the proportion in the levels of physical fitness between positive and negative responses in each correlate. Chi-square tests were used to evaluate the difference in the percentage of positive/negative outcomes of the single correlate in the survey questions and each of the five PFI groups with adjusted *p*-values (the Bonferroni method), if the significant difference existed in the preformation of chi-squared tests in the 2 × 5 design, with the positive/negative outcome of the survey item × each of the five PFI groups. Scatter plots were used to analyze the relationship between the total scores of ecological measures in each province and between high and low levels of PFI in children and adolescents by sex.

When analyzing the correlates of physical fitness with single correlate analysis, we then used logistic regression models, with an adjustment of age, sex, areas (urban and rural), province, and regional-level SES to assess the association between different levels of PFI and each correlates by positive responses and negative responses. In the regression models, the primary dependent outcome variables were different levels of physical fitness (separate high PFI, middle-high PFI, middle PFI, low-middle PFI, and low PFI), and the independent variables were 20 different correlates in the survey questions (positive vs. negative options), and the summary scores as continuous variables of 20 different correlates. Adjusted odds ratios (ORs), along with 95% confidence intervals, were presented. In the analysis of the multiple correlates, we assigned a value of 1 to a positive response (as a positive factor) and a negative value of −1 response (as a negative factor) to each of the 20 questionnaire items. We then derived a composite ecological score, representing a global index of the ecological correlate. We also examined the cumulative effects with both non-linear and linear associations between the global index (composite ecological score) and high and low level PFI. We employed generalized additive models with non-linear regression and logistic regression models with non-linear analyses to calculate the non-linear fitting curve and linear results with ORs of high or low PFI with a global index of ecological correlates (based on the median scores of “0”) in boys and girls, three age groups (10-12, 13-15, and 16-18 years), and three study regions. The three study regions were defined with 31 mainland Chinese provinces: East, Central, and West, in accordance with the geographical standard division from the Chinese National Bureau of Statistics (NBS) (30).

For further comparison of three independent ecological correlates of global index at individual, family, or school level, we set the same total score (all 1 point) in each level of ecological correlates, and high PFI was used as the response variable in a logistic regression analysis with summary scores as a continuous variable. The association between the summary scores within each independent ecological level (individual, family, and school) and the high levels of PFI was used to determine exactly which ecological level could be the strongest predictor of physical fitness with calculated OR. To understand the contribution of the three-level factors separately to the risk of high- and low-PFI levels, the population-attributable risks (PARs, %), with corresponding 95% confidence intervals, were estimated based on asymptotic approximations, which derived PARs, using the formula provided by Greenland and Drescher (31). Calculation of PAR implies a theoretical causal relationship and effect degree between three independent ecological correlates of global index at individual, family, or school level and high- or low-PFI levels. Models were conducted based on the logistic regression model, using the aflogit module for Stata adjusting for age, sex, region, and province. The theoretical high- or low-PFI-level proportions were assessed, using the actual rates multiplied by 1-PAR% if independent ecological correlates were eliminated or controlled, which reflected the intervention effects on PFI levels if three levels independent ecological correlates or all 20 ecological correlates were averted in children and adolescents theoretically (32). Statistical analyses were conducted with Stata (version 15.0) and R (version 3.5.1) statistical software. Two-sided *p* < 0.05 was considered statistically significant.

## Results

### Study Population Characteristics

The characteristics of the study population are presented in Supplementary Table 1. The proportion of sample sizes for the physical fitness and the questionnaire survey was balanced across sex, age, provinces, and urban and rural areas.

### Individual-, Family-, and School-Level Correlates of Physical Fitness

**Figure 1:**
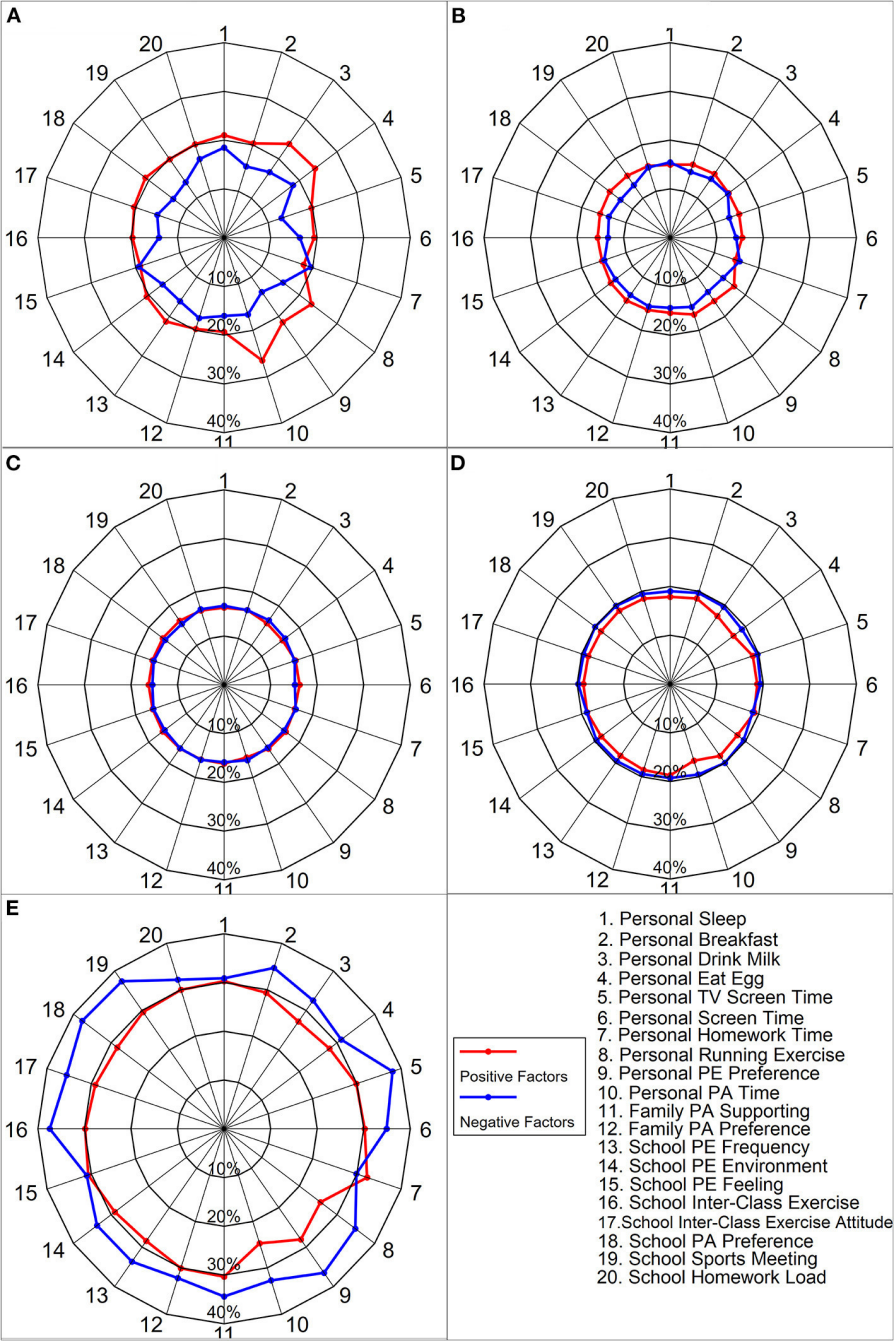
Differences in the five levels of physical fitness between positive and negative responses of 20 questions in questionnaire surveys. **(A)** High levels PFI. **(B)** High-middle levels PFI. **(C)** Middle levels PFI. **(D)** Low-middle levels PFI. **(E)** Low levels PFI. The number at the outer edge of the radar map represents the specific factors in the last legend subfigure. The number of percentages in the radar map represents the reference number of each line. For example, 40% represents the percentage of selected positive or negative responses of each question option (red line vs. blue line) outer line of the radar map. The statistically significant percentage difference between the positive and negative responses groups is presented in Supplementary Table 2.

As shown in radar maps in Figure 1, among the 20 questionnaire items that measured individual-, family-, and school-level correlates of physical fitness, 18 were shown to be significantly correlated with PFI. The two items that reflected individual homework time and daily experience at school PE showed no correlation with PFI (the exact statistical test is shown in Supplementary Table 2). Children and adolescents living in the eastern provinces showed a higher proportion of positive responses on the ecological correlates compared with those living in the central or western provinces (Supplementary Figure 1). In addition, as shown in Supplementary Figure 2, the positive responses of these ecological correlates were associated with a high proportion of high PFI, whereas negative responses were related to a low proportion of high PFI. The ORs estimates from the logistic regression analyses provided further support for this pattern, showing that positive responses of each correlate were more likely to be associated with children and adolescents who achieved high and middle-high PFI (Supplementary Table 3).

For example, children and adolescents who reported drinking at least 200- to 250-ml milk per day were associated with a higher proportion of high PFI (23.8%) than those who reported no milk consumption (16.6%) (OR = 1.47, 95% CI: 1.43, 1.51). In contrast, children and adolescents who reported no regular daily drink of 200- to 250-ml milk were associated with a higher proportion of low PFI (32.5%) than those who reported daily drinking (27.2%, OR: 1.27, 95% CI: 1.24, 1.30). With respect to PA, children who engaged in at least 1 h of daily PA were associated with a higher proportion of high PFI (26.5%) than those who did not (16.6%, OR: 1.65, 95% CI: 1.60, 1.70). Not meeting the recommended 1-h PA daily was significantly associated with a higher proportion of low PFI (32.7%) compared with meeting the recommendation (24.7%, OR: 1.44, 95% CI: 1.40, 1.48). Children and adolescents living in a supportive family or school environment for PA were associated with healthier physical fitness than those living in a less supportive environment for PA. For example, children and adolescents whose parents supported PA of their children or engaged in PA themselves were shown to be related to higher proportions of high PFI (19.3 and 19.7%) than those who did not (16.1 and 17.4%) with statistically significant ORs (1.22, 95% CI: 1.16, 1.28; and 1.10, 95% CI: 1.07, 1.13). Conversely, children and adolescents whose parents did not show support of PA of their children or engaged in PA themselves were associated with higher proportions of low PFI [34.40 vs. 30.35% with statistically significant ORs of 1.17 (1.12, 1.21); 32.20 vs. 30.08%, with statistically significant ORs of 1.07 (1.04, 1.10)] (Supplementary Table 2).

### Cumulative Effect of Ecological Correlates on Physical Fitness

**Figure 2:**
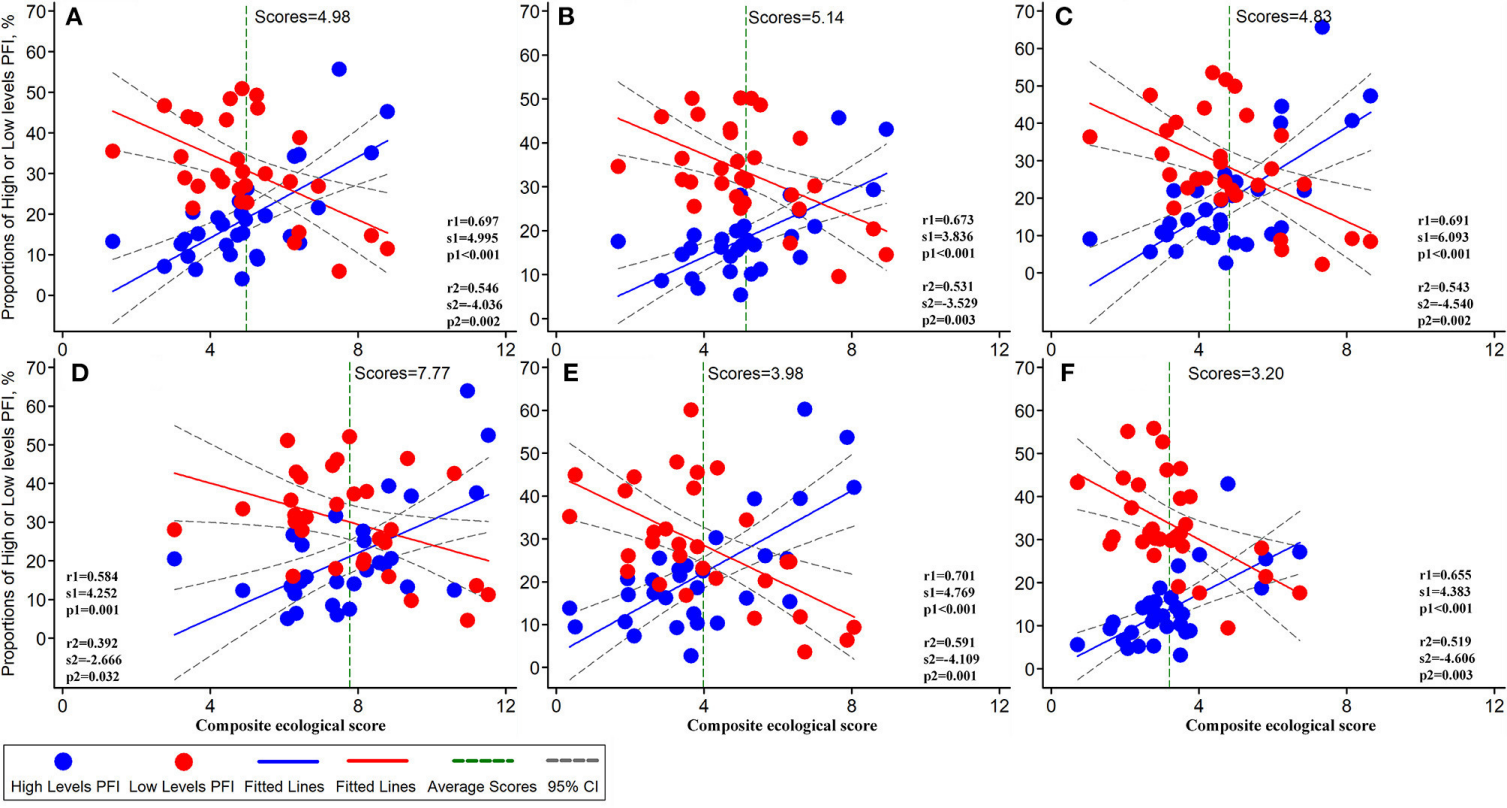
Distribution for proportions of high or low PFI with the scores of ecological correlates at the provincial level. **(A)** Total. **(B)** Boys. **(C)** Girls. **(D)** 10-12y. **(E)** 13-15y. **(F)** 16-18y. Each dot represents a province with the red dot for low-PFI levels and the blue dot for high PFI. The r represents a correlation coefficient, s denotes the slope rate (or rate of change) of the fitted curve, and the *p*-value indicates the level of statistical significance. Dotted green lines indicate the average scores of ecological correlates.

**Figure 3:**
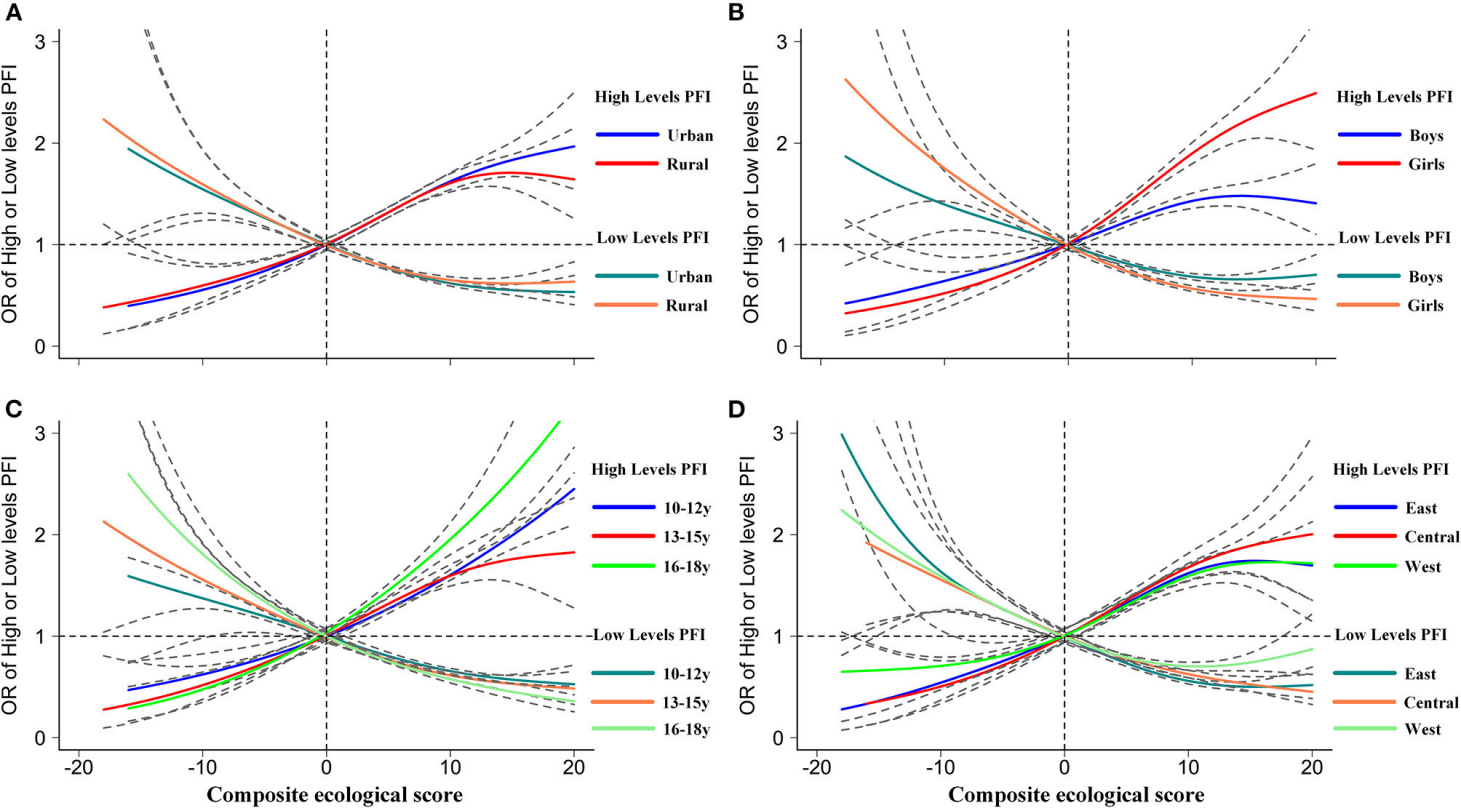
Subgroups analyses of the association between scores of ecological correlates and proportions of low- or high-PFI levels. **(A)** Areas. **(B)** Genders. **(C)** Age groups. **(D)** Regions. Dotted gray lines indicate 95% CIs. Generalized additive models were used to calculate the non-linear fitting curve of the OR values of high- or low-PFI levels (dependent outcome variables) with the scores of ecological correlates (as independent variables based on median scores of “0”).

Figure 2 presents a significant and positive correlation that existed between ecological correlates and provinces that had a high prevalence of high PFI and a negative correlation between the ecological correlates and provinces that showed a high prevalence of low PFI. Further subgroup analyses in Figure 3 and Supplementary Figure 3 show a significant and positive association between the ecological correlates and high PFI (adjusted ORs of 1.06, 95% CI: 1.06, 1.07) and a negative association between the ecological correlates and low PFI (adjusted ORs of 0.95, 95% CI: 0.95, 0.95) (Supplementary Table 4). Such positive and negative associations suggest that the multilevel ecological correlates at individual, family, and school levels had a significant cumulative effect on physical fitness among school children and adolescents. More positive responses on ecological correlates could contribute to the improvement of physical fitness, but more negative responses on ecological correlates could deteriorate physical fitness.

### The Separate Effect of Individual-, Family-, and School-Level Correlates on Physical Fitness

**Figure 4:**
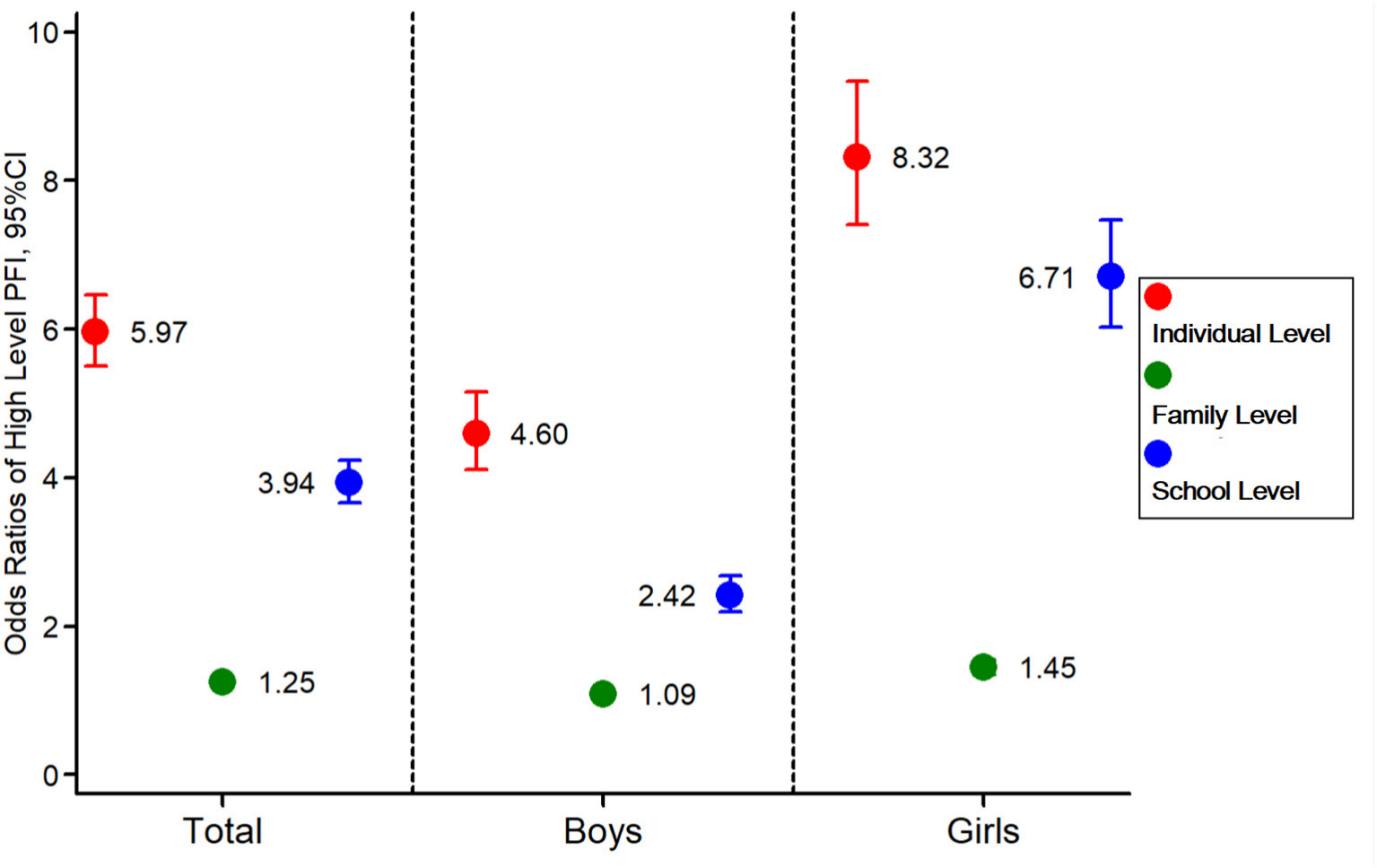
Comparison of the association strengths between high PFI and ecological correlates at an individual, family, or school level. The ORs assessing the associations between high-PFI levels (dependent outcome variables), and ecological correlates (independent variables) were calculated with adjustment for age, sex, area, province, and regional-level SES.

The population-attributable risks (%) were used to compare three levels of effect of ecological correlates on physical fitness, and the attribution and theoretical effect of control on physical fitness if independent ecological correlates were eliminated or controlled. Compared with the effects of family-level and school-level correlates (OR: 1.25, 95% CI: 1.19, 1.31; 3.94, 95% CI: 3.66, 4.24), the individual-level ecological correlates had the largest effect on the high PFI among Chinese children and adolescents (OR: 5.97, 95% CI: 5.51, 6.47). There was a similar association between high-PFI and individual-level correlates among boys and girls (Figure 4).

**Figure 5:**
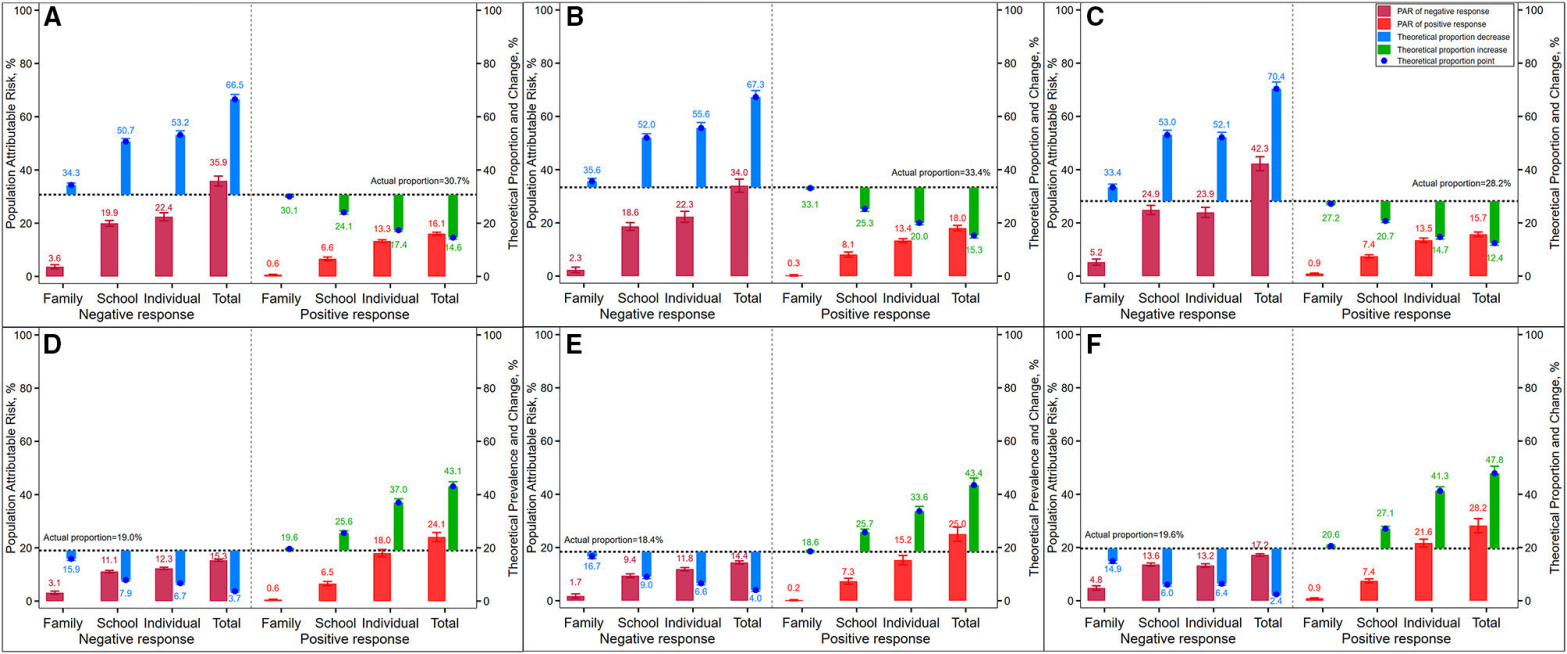
The population attributable risk and theoretical proportions of low- and high-PFI levels (dependent variables) and its theoretical changes to actual levels if 20 related influencing factors (independent variables) were controlled. **(A)** Total low PFI level. **(B)** Boys low PFI level. **(C)** Girls low PFI level. **(D)** Total high PFI level. **(E)** Boys high PFI level. **(F)** Girls high PFI level.

Figure 5 presents the PAR% for low and high PFI due to the multiple-level correlates. The theoretical low and high PFI proportions were also presented if negative or positive responses at multiple-level correlates were eliminated. The PAR% for low PFI due to the whole 20 correlates was 16.1-35.9%, and the theoretical low PFI proportion could increase to 66.5% or decrease to 14.6% based on the actual low-PFI proportion at 30.7% if all the positive or negative responses were eliminated separately. Similarly, the PAR% for high PFI due to all 20 correlates was 15.3-24.1%, and its theoretical high-PFI proportion could decrease to 3.7% or increase to 43.1% based on the actual high-PFI proportion at 19.% if these positive or negative responses at three multiple-level correlates were eliminated separately.

Individual level correlates were still the largest factor affecting physical fitness compared with the family-level and school-level correlates. For example, the control of individual-level negative correlates could pull down the theoretical high-PFI-level proportion from 19 to 6.7% due to its PAR% of 12.3%, followed by 11.1 and 3.1% of PAR% at family- and school-level correlates, respectively. However, the improvement of individual-level correlates with positive responses could pull up the theoretical high-PFI-level proportion from 19. to 43.1% due to its PAR% of 18.%, followed by 6.5 and.6% of PAR% at family- and school-level correlates, respectively.

## Discussion

To our knowledge, this is the first study that uses national representative survey data that contain a comprehensive fitness assessment of respiratory function, strength, flexibility, explosive power, and cardiorespiratory endurance functions. In such a cross-sectional survey involving over 150,000 students, we provided a global evaluation of individual-, family- and school-level correlates of physical fitness in child and adolescent populations. We found that the multilevel ecological correlates at individual, family, and school levels had a significant cumulative effect on physical fitness among school children and adolescents. In addition, we firstly took an integrated, ecological approach to identify that the correlates in the individual level were the most important factors affecting physical fitness, but they also need to be supported by correlates at school and family levels.

This study first highlighted the central role of individual-level correlates in physical improvement among children and adolescents, using national data. Considering that improving physical fitness at an individual level depends on individual PA, current international guidelines on PA for children and adolescents generally recommend positive lifestyles, including a high degree of participation in regular PA, a balanced diet, sufficient sleep, good personal hygiene, and avoidance of sedentary behaviors and harmful risks (e.g., smoking, drinking alcohol, taking drugs) (14, 33–35). Moreover, the reasonable allocation of individual daily time and healthy adjustment of lifestyle of children has become an important direction for the improvement of physical fitness, such as sleep rhythm, dietary patterns, the reasonable time management in daily PA, and sedentary behaviors. As the Canadian 24-h Movement Guidelines points out, allowing for the practical feasibility, the integration and balance of time spent per day in maintaining as many healthy behaviors as possible, including vigorous-intensity PA, sedentary behaviors, and sleep to enhance health outcomes among children and adolescents aged 5-17 years, should be promoted (34). The present study validated that individual ecological factors were central to improve physical fitness, which indicated the importance of increasing individual positive and healthy behaviors.

**Figure 6:**
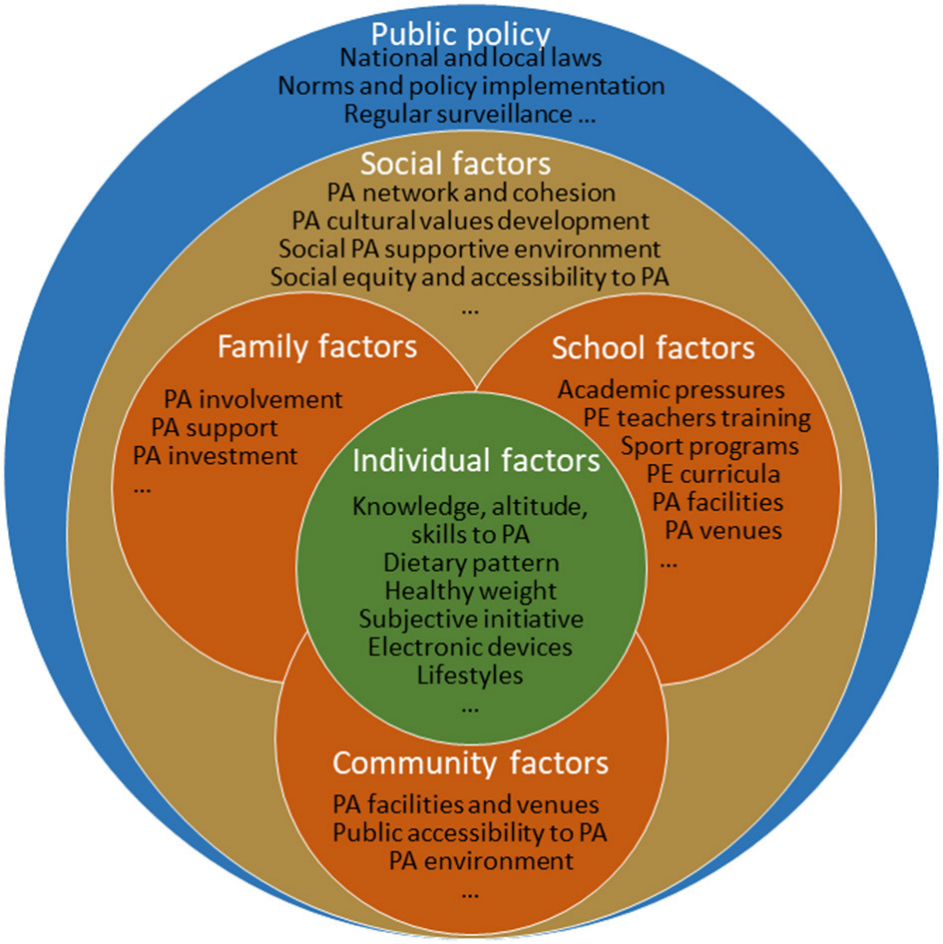
The multilevel correlates of physical fitness improvement among children and adolescents based on the social ecological model.

In addition to the core role of the correlates of the individual levels, our findings suggest family- and school-level correlates that are associated with the physical fitness of children and adolescents. In general, findings from our study are congruent with ecological theories and previous research, indicating that a socially supportive environment for PA at home and at school is likely related to the increased level of PA or physical fitness among children and adolescents (15, 16, 18, 22, 36). There is also consensus among the international community that positive family, community, and school engagement increases opportunities and promotes PA participation and improves the health of school-aged children and adolescents (21, 37–40). For example, the 2020 Physical Activity and Health Expert Consensus Statement in China indicates that environments supportive of physical exercise in the community, such as availability and accessibility of open space, recreational facilities, and walkable sidewalks, are likely to facilitate PA, reduce sedentary behaviors, and enhance physical fitness among children and adolescents (14). Thus, our findings continually support the importance for considering family, school, and community engagement in PA in setting school policies, implementing school PE classes or interventions, and enhancing physical fitness among school children and adolescents. Based on our social ecological model, as modeled in Figure 6, our findings might be further extended to multilevel interventions for physical fitness improvement among children and adolescents, including the individually centered factors improvement (PA, sleep, diet, sedentary behaviors, etc.), family, school, and community-PA-supportive environment built (availability and accessibility of open space, recreational facilities and walkable sidewalks, neighborhood design, etc.), improved socioeconomic factors, local and national policies (PA network and cohesion, regular surveillance, legislation, etc.).

From the health promotion perspective, priority areas in physical fitness programs need to be identified by integrating and implementing a wide comprehensive measure across individual, family, school, and community levels. Unsurprisingly, this study shows that health behavioral factors at the individual level remain at the core of physical fitness improvement among school children. However, supportive environmental improvements at the family, school, and community levels could also contribute to promoting the health of children through the individual level, such as by (a) policy and practice recommendations, (b) periodical physical fitness and health surveillance, (c) implementation of evidence-based health-related school PE curricula, (d) reductions in academic pressures, (e) staff qualifications and skill training of PE teachers and fitness instructors, (f) improvement of public accessibility to sports facilities and venues, and (g) community-wide promotion of after-school sport programs or school-community and school-family-linked PA programs. Apart from those multilevel efforts, strong health promotion measures at the individual level, such as the promotion of healthy lifestyles, breaking of sedentary time, engagement in sporting activities, and maintenance of normal weight, should also be encouraged and imposed on individual levels as a targeted measure.

There has been an increase in health-promotion policies at national and local levels. In 2007, the State Council of the Chinese government issued a strategic policy aimed at strengthening and enhancing physical fitness for school-aged children and adolescents (41). As an important landmark policy on physical fitness, it not only has made a positive impact on the health and well-being of children and adolescents but also has a significantly impactful influence on the health promotion policies and PA programs of the nation. In the years that followed, the government issued a total of 88 multisectoral measures and policies involving the promotion of sports, reductions in the academic burden through curriculum reform, and development of public facilities to promote the physical fitness of students (12, 42). Although there has been an increase in health-promotion policies in China, our findings highlight a notable geographic variation in the levels of physical fitness of children across regions and ecological correlates, as well as the significant association between them. These geographic disparities in physical fitness and ecological correlates may reflect an uneven development and implementation of nationwide policies, and the difference in the cumulative effect of multilevel ecological correlates in different regions. As such, in order to narrow these disparities across regions, integrated interventions and resources focused on individual, family, school, community, and policy environments built need to be implemented, particularly in the individual level, such as a child with poor physical fitness and places where resources were scarce. In addition, the targeted physical fitness interventions or programs should be directed toward school children by developing and promoting family-, school-, and community-level-PA-supportive environment that can maximize PA participation and physical fitness promotion with limitation of sedentary behaviors.

Our study has two notable strengths: First, we adopted a comprehensive approach to assessing physical fitness, which included respiratory function, strength, flexibility, explosive power, and cardiorespiratory endurance factions. Second, we assessed multilevel correlates of fitness that were derived from well-established ecological theories of PA in understanding the ecological factors that were associated with levels of physical fitness among children and adolescents. Despite these strengths, findings from our study should be interpreted in the context of several potential limitations. First, because the study used the data from a cross-sectional national survey, no causal inference can be inferred on our ecological analyses of the correlates associated with the levels of physical fitness, thus, nor should the findings be generalized to broader contexts without further causal study. Second, although this study employed multiple survey items assessing individual-, family-, and school-level correlates of physical fitness in children and adolescents, it is likely that we may have missed other important measures, such as features related to community-built environment, family- or individual-level SES, which may also explain trends, geographic variations, and levels of physical fitness in our study population. Third, instead of using the national fitness standards, we adopted a PFI approach to assessing physical fitness. However, such an approach is deemed appropriate in analyzing trends comparisons, and allows the categorization of age-related physical fitness data into different levels as we have operationalized. In addition, six physical fitness measures items of each child were completed almost in the same day, so it was possible exhaustion to them. The order of the different physical fitness measures items might affect the final physical fitness levels; thus, sensitivity test and analysis are needed in future surveys. Last but not the least, because of the use of survey data, recall bias, social desirability bias, and self-report bias are inevitable and can affect the validity and interpretation of our study outcomes. Due to the self-constructed questionnaire according to the realistic implementation, the lack of reliability and validity of data needs to be further evaluated. The exclusion of school dropouts, population mobility, and changes in population structure from 1985 to 2014 could also lead to the bias of regional sample representation.

## Conclusions

Findings from this study found a cluster of individual-, family- and school-level correlates that were associated with either improving or impeding the levels of physical fitness of children. Multilevel correlates at individual-, family- and school-multilevel factors had significant cumulative effects on the physical fitness of children. These findings suggest that the modifiable individual-, family-, and school-environmental factors are most likely to facilitate the improvement of physical fitness of children and youth across the country from diverse regions. The individual-level correlates are still the core of promoting the physical fitness of children, and schools and settings of families need to closely coordinate to ensure physical fitness improvement among children and adolescents. This study provides a direction for the policy priority formulation of physical fitness improvement in the future.

## Data Availability Statement

All the individual (de-identified) participant data collected in the surveys can be shared with investigators whose proposed use of the data has been approved by an independent review committee identified for this purpose by contacting the corresponding author. Proposals should be directed to majunt@bjmu.edu.cn and songyi@bjmu.edu.cn.

## Ethics Statement

The studies involving human participants were reviewed and approved by Medical Research Ethics Committee of the Peking University Health Science Center (IRB00001052-13002). Written informed consent to participate in this study was provided by the participants' legal guardian/next of kin.

## Author Contributions

YD conceptualized and designed the study, completed the statistical analyses, drafted the initial manuscript, and reviewed and revised the manuscript. JM and YS contributed to the conceptualization and design of the study, supervised the data collection, the statistical analyses and initial drafting of the manuscript, and reviewed and revised the manuscript. PL participated in conceiving the study design and critically reviewed and revised the manuscript from the preliminary draft to submission. MC, LC, YY, ZW, BW, and YM assisted with data interpretation and reviewed and revised the manuscript. All authors approved the final manuscript as submitted and agreed to be accountable for all aspects of the work.

## Conflict of Interest

The authors declare that the research was conducted in the absence of any commercial or financial relationships that could be construed as a potential conflict of interest.

## Publisher's Note

All claims expressed in this article are solely those of the authors and do not necessarily represent those of their affiliated organizations, or those of the publisher, the editors and the reviewers. Any product that may be evaluated in this article, or claim that may be made by its manufacturer, is not guaranteed or endorsed by the publisher.
