# Supplementary material for: Individual-, Family-, and School-Level Ecological Correlates With Physical Fitness Among Chinese School-Aged Children and Adolescents: A National Cross-Sectional Survey in 2014
Source: Front Nutr. 2021 Aug 25;8:684286. doi: 10.3389/fnut.2021.684286 (PMC8424096; doi:10.3389/fnut.2021.684286)
Supplement: Supplementary file 1 [file Data_Sheet_1.DOCX]

Supplementary Material


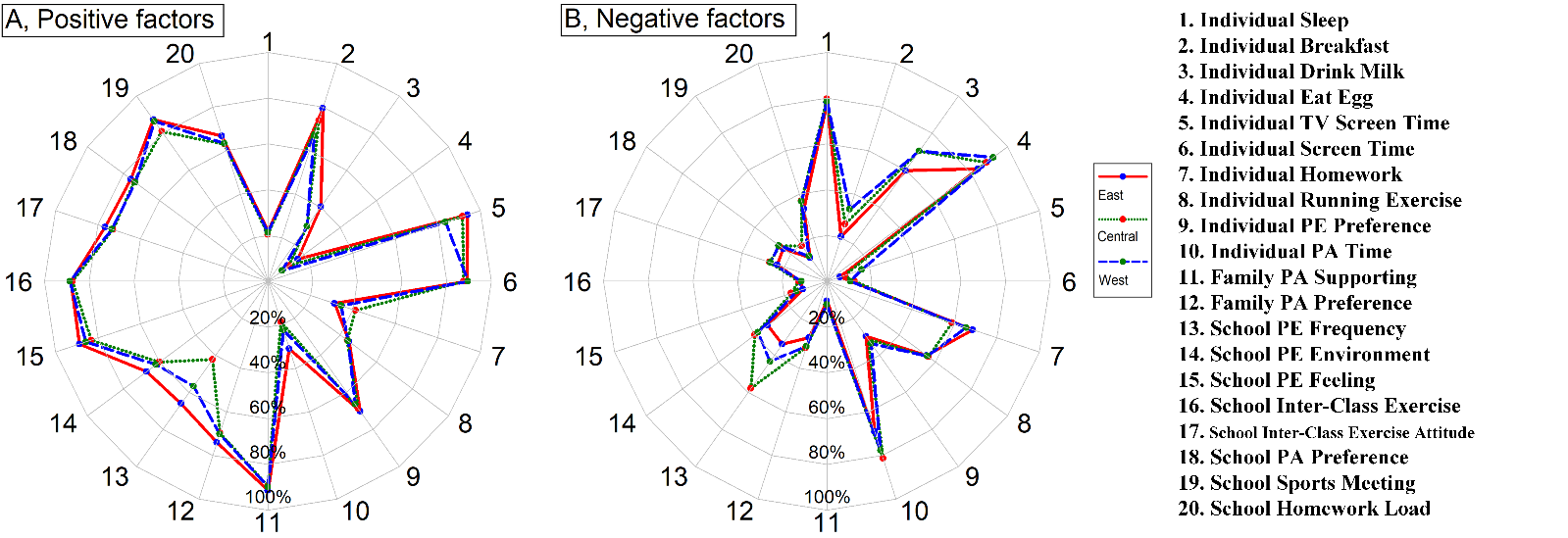


**Figure S1.** The proportions of the positive and negative factors in eastern, central and western regions

Note: The number at the outer edge of a radar map represents the specific factors in the right legend subfigure. The number of percentage in the radar map represents the reference number of each line, for example, 40% represents the percentage of selected positive or negative factor of each question option of outer line of radar map.


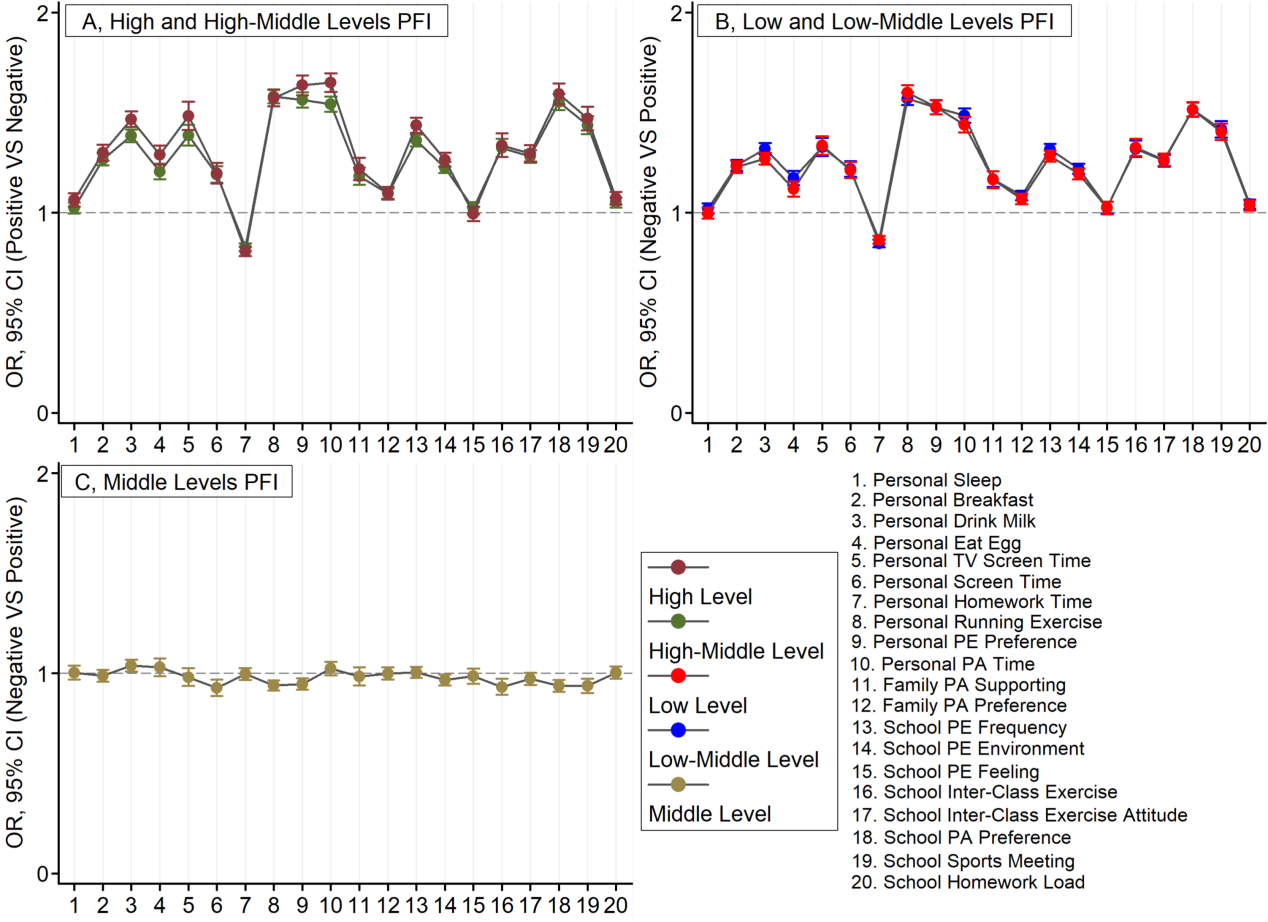


**Figure S2.** The ORs of five levels of physical fitness between the positive and negative factors based on the different dimensions in questionnaire surveys

Note: The ORs were calculated after adjusting the age, sex, region and province using the Logistic regression models with the reference group (Positive Factors v.s. Negative Factors in High PFI and Middle-High PFI; Negative Factors v.s. Positive Factors in Low PFI, Low-Middle PFI and Middle PFI)*. In the logistic regression analysis, the dependent outcome variables were whether High PFI and Middle-High PFI was or not (Figure A)*, and whether Low PFI, Low-Middle PFI and Middle PFI was or not (Figure B and C)*, while the independent variable is whether children chose the positive factor (Figure A)* and negative factor (Figure B and C)* in each survey item or correlate (20 items)*.


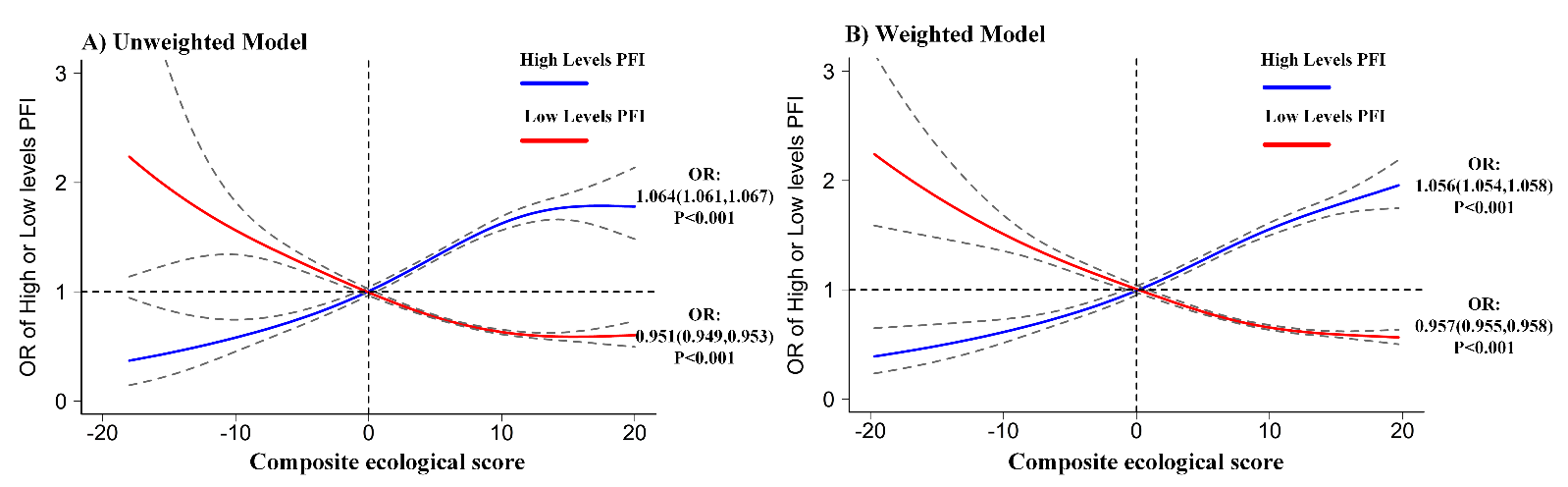


**Figure S3.** The association between scores of comprehensive influencing factors and low or high PFI levels using unweighted and weighted model

Note: Dotted gray lines indicate 95% CIs. Generalized additive models were used to calculate the non-linear fitting curve of the OR values of high or low PFI levels with global index of ecological correlates (based on median scores of “0”)*. The exact OR values were calculated using the Logistic regression model after adjusting the age, sex, area, province and regional level socioeconomic status (SES)*. Sensitivity analysis was performed by comparing the results of the weighted and unweighted models. In the unweighted model, we set the same weight coefficient of each factor affecting physical fitness. In the weighted model, we set the regression coefficient in regression analysis between each factor and outcome variables (physical fitness)* as the weighting coefficient.

| **Table S1.** The sample size and percentage of anthropometric and physical fitness measurements, in 2014 | |
| --- | --- |
| **Survey year** | **2014** |
| **Total sample size** | 157168 |
| **Gender, n(%)*** | |
| Boys | 77878(49.6)* |
| Girls | 79290(50.4)* |
| **Areas, n(%)*** | |
| Urban | 78208(49.8)* |
| Rural | 78960(50.2)* |
| **Age groups, n(%)*** | |
| 10-12y | 52481(33.4)* |
| 13-15y | 52456(33.4)* |
| 16-18y | 52230(33.2)* |
| **Anthropometric measurement, n (%)*** | |
| Height | 157168(100.00)* |
| Weight | 157168(100.00)* |
| **Physical fitness measurement, n (%)*** | |
| Forced Vital Capacity | 157168(100.00)* |
| Standing Long Jump | 157168(100.00)* |
| Sit and Reach | 157168(100.00)* |
| 50 Meter Dash | 157168(100.00)* |
| Body Muscle Strength | 157168(100.00)* |
| Endurance running | 157168(100.00)* |
| Note: n (%)*, the sample size and percentage completing each physical fitness measurement. | |

| **Table S2**. Description of variables and categories of the positive and negative options | | | | | | | |
| --- | --- | --- | --- | --- | --- | --- | --- |
| Questions | Options | Sample | PFI Proportions | | | | |
|  |  |  | low | low-middle | middle | middle-high | high |
| **Q1:(Sleep)* In the past year, what was your average sleep time per day?** § **(Response rate 100%)*** | | | | | | | |
| Positive: | Sufficiency | 33,490 | 30.29 | 17.88* | 15.84 | 14.97* | 21.02* |
| Negative: | Insufficiency | 123,678 | 30.87 | 19.02 | 16.20 | 15.44 | 18.47 |
| **Q2:(Breakfast)*In the past year, whether to eat breakfast every day? (Response rate 99.88%)*** | | | | | | | |
| Positive | Yes | 115,201 | 29.32* | 18.46* | 16.13 | 15.76* | 20.33* |
| Negative | No | 41,778 | 34.7 | 19.65 | 16.11 | 14.18 | 15.36 |
| **Q3:(Drink Milk)*In the past year, did you drink at least one bag of milk per day? (One bag represents the amount of milk 200-250 ml or 200-250 g)* (Response rate 100%)*** | | | | | | | |
| Positive | Yes | 52,741 | 27.19* | 17.24* | 15.61 | 16.17* | 23.78* |
| Negative | No | 104,427 | 32.54 | 19.56 | 16.38 | 14.92 | 16.61 |
| **Q4:(Eat Egg)*In the past year, did you eat at least one egg per day? (Response rate 99.87%)*** | | | | | | | |
| Positive | Yes | 18,871 | 28.00* | 16.81* | 15.52 | 15.51 | 24.16* |
| Negative | No | 138,099 | 31.12 | 19.05 | 16.21 | 15.32 | 18.30 |
| **Q5:(TV Screen Time)*In the past year, what was your average daily time watching TV? (Response rate 100%)*** | | | | | | | |
| Positive | < 2h | 88,349 | 29.88* | 18.67* | 16.14 | 15.58* | 19.72* |
| Negative | ≥2h | 68,819 | 38.06 | 19.71 | 15.95 | 13.32 | 12.96 |
| **Q6:(Screen Time)*In the past year, what was your average daily time spending playing games, reading video or reading e-books on your phone, tablet, computer, or video game console, etc? (Responserate 100%)*** | | | | | | | |
| Positive | < 2h | 107,516 | 30.22* | 18.70* | 16.24 | 15.49* | 19.36* |
| Negative | ≥2h | 49,652 | 34.94 | 19.40 | 15.20 | 14.18 | 16.28 |
| **Q7:(Homework Time)*In the past year, what was the average daily time you spend on homework? (Response rate 99.87%)*** | | | | | | | |
| Positive | < 1h | 99,481 | 32.32* | 19.03* | 15.98 | 14.70* | 17.97* |
| Negative | ≥1h | 57,477 | 29.89 | 18.64 | 16.20 | 15.69 | 19.58 |
| **Q8:(Running Exercise)*Would you like to take part in long-distance running exercise? (Response rate 100%)*** | | | | | | | |
| Positive | Yes | 135,316 | 25.61* | 17.84* | 16.39 | 16.96* | 23.20* |
| Negative | No | 21,852 | 34.85 | 19.53 | 15.91 | 14.05 | 15.67 |
| **Q9:(PE Preference)*Do you like physical education class? (Response rate 100%)*** | | | | | | | |
| Positive | Yes | 138,481 | 28.37* | 18.38* | 16.28 | 16.07* | 20.90* |
| Negative | No | 18,687 | 37.67 | 19.95 | 15.65 | 13.21 | 13.52 |
| **Q10:(PA Time)*In the past year, what was the average daily time you spend on physical activities? (Response rate 100%)*** | | | | | | | |
| Positive | ≥1h | 133,951 | 24.71* | 16.53* | 15.67 | 16.58* | 26.50* |
| Negative | < 1h | 23,217 | 32.70 | 19.51 | 16.27 | 14.94 | 16.58 |
| **Q11:(Family PA Supporting)*Do your parents support you to participate in physical activities in your spare time? (Response rate 99.85%)*** | | | | | | | |
| Positive | Yes | 116,884 | 30.35* | 18.72* | 16.15 | 15.44 | 19.33* |
| Negative | No | 40,045 | 34.40 | 19.29 | 15.87 | 14.40 | 16.05 |
| **Q12:(Family PA Preference)*Do your parents like to take physical exercise in their spare time? (Response rate 100%)*** | | | | | | | |
| Positive | Yes | 116,999 | 30.08* | 18.53* | 16.11 | 15.58 | 19.69* |
| Negative | No | 40,169 | 32.20 | 19.42 | 16.18 | 14.84 | 17.36 |
| **Q13:(School PE Frequency)*How many standard classes did you take per week in physical education last semester?# (Response rate 100%)*** | | | | | | | |
| Positive | Sufficiency | 38,543 | 28.44* | 18.19* | 16.15 | 15.95 | 21.26* |
| Negative | Insufficiency | 118,625 | 33.69 | 19.54 | 16.09 | 14.56 | 16.12 |
| **Q14:(School PE Environment)*Does your school have the phenomenon that occupy or do not offer physical education? (Response rate 100%)*** | | | | | | | |
| Positive | No | 141,992 | 29.02* | 18.31* | 16.31 | 15.80 | 20.56* |
| Negative | Yes | 15,176 | 33.74 | 19.59 | 15.79 | 14.54 | 16.34 |
| **Q15:(School PE Feeling)*** **Do you feel tired from gym class at school (Response rate 99.28%)*** | | | | | | | |
| Positive | Sweaty/Tired | 111,434 | 30.70 | 18.77 | 16.15 | 15.41 | 18.97 |
| Negative | No sweat/Relaxed | 44,602 | 31.03 | 18.85 | 15.96 | 14.90 | 19.27 |
| **Q16:(School Inter-Class Exercise)*In the past year, how many times did you take school inter-class exercise per day? (Response rate 100%)*** | | | | | | | |
| Positive | ≥Once | 69,837 | 29.84* | 18.65* | 16.22 | 15.61* | 19.69* |
| Negative | No | 87,331 | 37.46 | 19.76 | 15.41 | 13.37 | 13.99 |
| **Q17:(School Inter-Class Exercise Attitude)*In the past year, did you take school inter-class exercise seriously? (Response rate 100%)*** | | | | | | | |
| Positive | Go every time/take it seriously | 101,321 | 29.10* | 18.51* | 16.2 | 15.84* | 20.35* |
| Negative | Often absent/indifference | 55,847 | 35.58 | 19.56 | 15.89 | 13.87 | 15.10 |
| **Q18:(School PA Preference)*Do you like to take part in extracurricular sports organized by the school? (Response rate 100%)*** | | | | | | | |
| Positive | Yes | 55,206 | 28.07* | 18.22* | 16.22 | 16.07* | 21.43* |
| Negative | No | 101,962 | 36.53 | 20.00 | 15.92 | 13.77 | 13.78 |
| **Q19:(School Sports Competition)*How many sports meetings have the school organized in recent academic year? (Response rate 100%)*** | | | | | | | |
| Positive | ≥Once | 140,656 | 29.59* | 18.60* | 16.24 | 15.70* | 19.87* |
| Negative | No | 16,512 | 37.39 | 19.81 | 15.44 | 13.30 | 14.06 |
| **Q20:(School Homework Load)*How do you think about the school homework load? (Response rate 100%)*** | | | | | | | |
| Positive | Fair enough to handle | 139,636 | 29.97* | 18.43 | 16.02 | 15.44 | 20.14* |
| Negative | Heavy, laborious, or unable to cope | 17,532 | 32.15 | 19.41 | 16.31 | 15.16 | 16.97 |
| Note: §According to China's regulations on school health, primary school students should get more than 10 hours of sleep per day, junior high school students should get at least 9 hours and senior high school students should get at least 8 hours. #According to China's education class hour regulations, students in primary and middle schools need three standard class hours of physical education per week, and those in high schools need two standard class hours of physical education per week. * Represents the percentage difference between the two groups was statistically significant using the chi-square tests. Chi-square tests were used to evaluate the difference in percentage of positive/negative outcome of the single correlate in the survey questions and each of the 5 PFI groups with a adjust P values (Bonferroni method)*, if the significant difference existed in the preformation of chi-squared tests in the 2×5 design with the positive/negative outcome of the survey item × each of the 5 PFI groups (*P*<0.05)*. | | | | | | | |

| **Table S3**. The ORs of the positive factors answers from the questionnaires for high and middle-high levels of PFI, and the ORs of negative factors' answers from the questionnaires for low, low-middle and middle levels of PFI | | | | | | |
| --- | --- | --- | --- | --- | --- | --- |
| Factors | Positive Factors | |  | Negative Factors | | |
|  | High PFI | Middle-High PFI | | Low PFI | Low-Middle PFI | Middle PFI |
| **Individual factors** |  |  |  |  |  |  |
| Individual Sleep | 1.06(1.03,1.10)* | 1.02(1.00,1.05)* |  | 1.00(0.97,1.03) | 1.02(0.99,1.05) | 1.00(0.97,1.04) |
| Individual Breakfast | 1.30(1.26,1.34)* | 1.27(1.24,1.30)* |  | 1.23(1.20,1.26)* | 1.24(1.21,1.27)* | 0.99(0.96,1.02) |
| Individual Drink Milk | 1.47(1.43,1.51)* | 1.38(1.35,1.42)* |  | 1.27(1.24,1.30)* | 1.32(1.29,1.35)* | 1.04(1.01,1.07)* |
| Individual Eat Egg | 1.29(1.24,1.34)* | 1.21(1.17,1.25)* |  | 1.12(1.08,1.16)* | 1.17(1.14,1.21)* | 1.03(0.99,1.07) |
| Individual TV Screen Time | 1.48(1.41,1.56)* | 1.39(1.34,1.44)* |  | 1.34(1.29,1.38)* | 1.33(1.28,1.37)* | 0.98(0.94,1.03) |
| Individual Screen Time | 1.20(1.15,1.25)* | 1.19(1.15,1.23)* |  | 1.21(1.17,1.25)* | 1.22(1.18,1.26)* | 0.93(0.89,0.97)* |
| Individual Homework Time | 0.81(0.78,0.83)* | 0.83(0.81,0.85)* |  | 0.86(0.84,0.89)* | 0.85(0.83,0.87)* | 1.00(0.97,1.03) |
| Individual Running Exercise | 1.57(1.53,1.61)* | 1.58(1.55,1.62)* |  | 1.60(1.56,1.64)* | 1.57(1.54,1.60)* | 0.94(0.91,0.97)* |
| Individual PE Preference | 1.64(1.59,1.69)* | 1.56(1.53,1.60)* |  | 1.53(1.49,1.56)* | 1.53(1.49,1.56)* | 0.95(0.92,0.98)* |
| Individual PA Time | 1.65(1.60,1.70)* | 1.54(1.51,1.58)* |  | 1.44(1.40,1.48)* | 1.49(1.45,1.52)* | 1.02(0.99,1.06) |
| **Family factors** |  |  |  |  |  |  |
| Family PA Supporting | 1.22(1.16,1.28)* | 1.18(1.14,1.23)* |  | 1.17(1.12,1.21)* | 1.17(1.13,1.21)* | 0.98(0.94,1.03) |
| Family PA Preference | 1.10(1.07,1.13)* | 1.10(1.07,1.12)* |  | 1.07(1.04,1.10)* | 1.09(1.06,1.11)* | 1.00(0.97,1.03) |
| **School factors** |  |  |  |  |  |  |
| School PE Frequency | 1.44(1.40,1.48)* | 1.36(1.33,1.39)* |  | 1.28(1.26,1.31)* | 1.32(1.29,1.34)* | 1.00(0.98,1.03) |
| School PE Environment | 1.27(1.23,1.30)* | 1.23(1.20,1.25)* |  | 1.19(1.17,1.22)* | 1.22(1.20,1.25)* | 0.97(0.94,0.99)* |
| School PE Feeling | 0.99(0.96,1.03) | 1.02(0.99,1.05) |  | 1.02(0.99,1.06) | 1.03(1.00,1.06)* | 0.99(0.95,1.02) |
| School Inter-Class Exercise | 1.34(1.28,1.40)* | 1.32(1.28,1.37)* |  | 1.33(1.28,1.37)* | 1.32(1.28,1.36)* | 0.93(0.89,0.97)* |
| School Inter-Class Exercise Attitude | 1.30(1.26,1.34)* | 1.28(1.25,1.31)* |  | 1.27(1.23,1.30)* | 1.26(1.23,1.29)* | 0.97(0.94,1.00) |
| School PA Preference | 1.59(1.54,1.65)* | 1.55(1.51,1.59)* |  | 1.52(1.48,1.55)* | 1.52(1.48,1.55)* | 0.94(0.91,0.97)* |
| School Sports Competition | 1.47(1.41,1.53)* | 1.44(1.39,1.48)* |  | 1.40(1.36,1.45)* | 1.42(1.38,1.46)* | 0.94(0.90,0.97)* |
| School Homework Load | 1.07(1.04,1.10)* | 1.05(1.03,1.08)* |  | 1.04(1.01,1.06)* | 1.04(1.02,1.07)* | 1.00(0.97,1.03) |

Note: * indicated the statistically significant difference with P values less than 0.05. The ORs were calculated after adjusting the age, sex, region and province using the Logistic regression models with the reference group (Positive Factors v.s. Negative Factors in High PFI and Middle-High PFI; Negative Factors v.s. Positive Factors in Low PFI, Low-Middle PFI and Middle PFI). In the logistic regression analysis, the dependent outcome variables were whether High PFI and Middle-High PFI was or not (Figure A), and whether Low PFI, Low-Middle PFI and Middle PFI was or not (Figure B and C), while the independent variable is whether children chose the positive factor (Figure A) and negative factor (Figure B and C) in each survey item or correlate (20 items).

| **Table S4.** The ORs of high PFI levels or low PFI levels in different percentiles groups of scores of comprehensive influence factors | | | | | |
| --- | --- | --- | --- | --- | --- |
| Groups | Percentiles groups of scores of comprehensive influence factors | | | | |
|  | 5th | 25th | 50th | 75th | 95th |
| High levels PFI | |  |  |  |  |
| Total | 0.40(0.19,0.88)* | 0.62(0.51,0.75)* | 1.05(1.02,1.09)* | 1.64(1.58,1.71)* | 1.79(1.57,2.03)* |
| Genders |  |  |  |  |  |
| Boys | 0.46(0.19,1.14)* | 0.68(0.53,0.87)* | 1.04(0.99,1.10)* | 1.44(1.36,1.52)* | 1.44(1.21,1.72)* |
| Girls | 0.36(0.14,0.91)* | 0.56(0.43,0.73)* | 1.07(1.02,1.12)* | 1.93(1.83,2.05)* | 2.41(2.01,2.89)* |
| Age groups | |  |  |  |  |
| 10-12y | 0.50(0.33,0.77)* | 0.71(0.57,0.88)* | 1.09(1.03,1.16)* | 1.67(1.61,1.73)* | 2.27(2.02,2.56)* |
| 13-15y | 0.31(0.13,0.77)* | 0.57(0.44,0.72)* | 1.06(1.01,1.11)* | 1.61(1.51,1.73)* | 1.81(1.39,2.34)* |
| 16-18y | 0.33(0.20,0.53)* | 0.60(0.49,0.73)* | 1.18(1.14,1.22)* | 2.06(1.87,2.26)* | 3.01(2.27,4.00)* |
| Regions |  |  |  |  |  |
| East | 0.32(0.11,0.96)* | 0.59(0.44,0.80)* | 1.06(1.00,1.12)* | 1.64(1.56,1.72)* | 1.72(1.47,2.02)* |
| Central | 0.37(0.16,0.86)* | 0.61(0.48,0.77)* | 1.12(1.06,1.19)* | 1.73(1.58,1.88)* | 1.97(1.47,2.63)* |
| West | 0.66(0.20,2.11)* | 0.72(0.53,0.99)* | 1.05(0.99,1.12)* | 1.61(1.49,1.74)* | 1.73(1.29,2.31)* |
| Low levels PFI | |  |  |  |  |
| Total | 2.08(1.20,3.61)* | 1.48(1.32,1.67)* | 0.95(0.93,0.98)* | 0.62(0.60,0.65)* | 0.59(0.52,0.68)* |
| Genders |  |  |  |  |  |
| Boys | 1.76(0.87,3.55)* | 1.34(1.14,1.58)* | 0.96(0.92,1.00)* | 0.68(0.65,0.71)* | 0.68(0.65,0.71)* |
| Girls | 2.43(1.24,4.75)* | 1.65(1.40,1.93)* | 0.94(0.90,0.98)* | 0.56(0.53,0.59)* | 0.48(0.39,0.58)* |
| Age groups | |  |  |  |  |
| 10-12y | 1.54(0.80,2.95)* | 1.28(1.03,1.58)* | 0.93(0.87,0.98)* | 0.64(0.61,0.66)* | 0.54(0.46,0.63)* |
| 13-15y | 2.01(1.11,3.63)* | 1.48(1.26,1.74)* | 0.95(0.91,1.00)* | 0.61(0.57,0.65)* | 0.50(0.37,0.67)* |
| 16-18y | 2.38(1.72,3.29)* | 1.54(1.37,1.72)* | 0.90(0.88,0.93)* | 0.55(0.51,0.60)* | 0.39(0.30,0.51)* |
| Regions |  |  |  |  |  |
| East | 2.63(1.04,6.66)* | 1.53(1.23,1.89)* | 0.95(0.89,1.01)* | 0.55(0.52,0.58)* | 0.51(0.41,0.63)* |
| Central | 1.83(1.16,2.89)* | 1.40(1.22,1.61)* | 0.91(0.88,0.95)* | 0.60(0.57,0.65)* | 0.48(0.37,0.61)* |
| West | 2.10(0.91,4.82)* | 1.51(1.26,1.81)* | 0.95(0.91,1.00)* | 0.70(0.66,0.74)* | 0.81(0.64,1.04)* |
| Note: Generalized additive models were used to calculate the non-linear fitting curve of the OR values of high or low PFI levels with the scores of comprehensive influencing factors (based on median scores of “0”)* after adjusting the age, sex, region and province. | | | | | |
